# Noncompliance with the WHO's Recommended Eight Antenatal Care Visits among Pregnant Women in Sub-Saharan Africa: A Multilevel Analysis

**DOI:** 10.1155/2021/6696829

**Published:** 2021-09-17

**Authors:** Emmanuel Kolawole Odusina, Bright Opoku Ahinkorah, Edward Kwabena Ameyaw, Abdul-Aziz Seidu, Eugene Budu, Betregiorgis Zegeye, Sanni Yaya

**Affiliations:** ^1^Department of Demography and Social Statistics, Faculty of Social Sciences, Federal University, Oye Ekiti, Nigeria; ^2^The Australian Centre for Public and Population Health Research (ACPPHR), Faculty of Health, University of Technology, Sydney, Australia; ^3^Department of Population and Health, University of Cape Coast, Cape Coast, Ghana; ^4^College of Public Health, Medical and Veterinary Sciences, James Cook University, Townsville, Queensland, Australia; ^5^HaSET Maternal and Child Health Research Program, Addis Ababa, Ethiopia; ^6^University of Parakou, Faculty of Medicine, Parakou, Benin

## Abstract

**Background:**

In 2016, the World Health Organization (WHO) introduced a minimum of eight antenatal care (ANC) visits for positive pregnancy outcomes. This study examined the prevalence of noncompliance with 8+ ANCvisits and its associated factors in sub-Saharan Africa (SSA).

**Methods:**

We used data from the Demographic and Health Surveys of eight countries in SSA. A pooled sample of 63,266 pregnant women aged 15-49 years who had given birth to children within 5 years prior to the surveys was included in this study. To examine the factors associated with noncompliance with ANC 8+ visits, multilevel binary logistic regression analysis was conducted, and the results were reported using odds radios at 95% confidence interval (CI).

**Results:**

The pooled prevalence of noncompliance with ANC 8+ visits was 92.3% (95% CI: 91.1%-93.3%) with the highest and lowest prevalence in Zambia (98.7%, 95% CI: 98.3%-99.1%) and Libya (73.4%, 95% CI: 70.4%-76.2%), respectively. With the individual level factors, women's age (44-49 years-aOR = 0.33, 9% CI: 0.14-0.78), health insurance registration, (yes-aOR = 0.53, 95% CI: 0.29-0.98), and economic status (richest-aOR = 0.16, 95% CI: 0.05-0.49) were negatively associated with noncompliance with 8+ ANC visits, while parity (five or more children-aOR = 1.68, 95% CI: 1.12-2.52) was positively associated with noncompliance with 8+ ANC visit. With the community level factors, community level literacy was negatively associated with noncompliance with 8+ ANC visit (high-aOR = 0.56, 95% CI: 0.32-0.99).

**Conclusion:**

About eight out of ten pregnant women did not comply with the WHO's recommended minimum of eight ANC visits for positive pregnancy outcomes in SSA. Empowering the economic status of women , enhancing health insurance and education coverage, and giving more attention to young pregnant women and those with more children are crucial for improving the coverage of ANC 8+ visits in the region.

## 1. Background

Globally, efforts toward achieving significant reduction in maternal and child morbidity and mortality have not achieved desired outcome, especially in low- and middle-income countries (LMICs) [1–3]. Though ANC services have been associated with decrease in maternal morbidity and mortality [4, 5], overall, about five out of every ten women in LMICs do not receive adequate ANC services [6, 7]. A significant proportion of women of reproductive age die due to pregnancy and childbirth complications daily [1, 2], with more burden in LMICs [4]. About 99 percent of estimated maternal deaths in 2015 occurred in LMICs [2, 8], and it is more pronounced in sub-Saharan Africa (SSA) [2].

For better pregnancy outcome, the use of ANC services is a key component [1, 4, 7]. This is associated with the rate of maternal morbidity and mortality [1, 4]. Also, paramount to better pregnancy outcome is the number of ANC visits [1, 8]. To achieve the global Sustainable Development Goals (SDG) especially the third goal of achieving a reduction in maternal mortalities to less than 70 per 100,000 live births by 2030, evidence-based interventions and policies are required [4, 7, 9].

The World Health Organization (WHO) came up with a policy of eight aANCvisits for pregnant women in 2016 [10], due to high incidence of maternal mortality worldwide, especially in LMICs [10]. To ensure comprehensive healthcare during pregnancy and childbirth, the number of ANC visits was increased from four to eight [10]. Findings show that higher frequency of antenatal attendance is related to reduce odds of stillbirths and eight ANC visits can reduce perinatal mortality to eight per 1,000 births [10]. Higher frequency of visits ensures delivery care and timely information to pregnant women and help in the identification and prevention of adverse pregnancy consequences [10–12].

To reduce maternal and childhood morbidity and mortality, the use of maternal healthcare services cannot be overemphasised [7, 13, 14]. Appropriate ANC visits have been linked to reduction in maternal morbidity and mortality [7]. Studies have linked the utilization of healthcare facility with ANC attendances [10, 13, 15–18]. To reduce pregnancy and childbirth complications, the number of ANC visits during pregnancy is vital [11, 12, 19]. Evidence showed that 70% of the indirect causes of maternal mortality were the result of prior disorders aggravated by pregnancy [20]; therefore, the role of ANC services is of immerse value for positive pregnancy outcome [11]. Also, evidence from the literature has revealed poverty as a factor responsible for women's inability to seek appropriate medical attention and care [21]. Other factors in maternal healthcare service utilization are education, income, mass media, residence, and work status [4].

Despite different efforts to reduce maternal morbidity and mortality, still, many women are dying from preventable pregnancy and childbirth complication [10]. Hence, the WHO designed a new policy: eight and above ANC visits [10]. Available studies have discussed the importance of ANC services [12, 19] and have emphasised the importance of demand and supply sides of ANC [11, 12]. However, there is a dearth of evidence on the prevalence of noncompliance with the WHO's recommended eight ANC visits as well as its associated factors in SSA. This study, therefore, examined noncompliance with the new WHO recommended eight ANC visits and its associated factors in eight countries in SSA.

## 2. Methods

### 2.1. Data Source

Data were derived from nationally representative Demographic and Health Surveys (DHSs) of eight SSA countries, which had information on birth histories. The study explored the DHS datasets spanning 2018 to 2020, and all women aged between 15-49 years who reported at least one birth in the five years preceding the surveys were considered. The guideline was launched in 2016, and the data collected in DHS included five years preceding the survey; as a result, DHS before 2016 and one year after launching the guideline (2017) is not included because immediate implementation was not possible due to the fact that time is needed for adoption of the guideline and related preparation including training. DHS data are collected usually every five years with the use of pretested validated quantitative tools and structured methodologies. The multicountry analysis is possible due to consistency of the method of survey over time and across countries. Including ANC services, data are collected on a wide range of public health-related issues such as demographic, socioeconomic, anthropometric, maternity history, family planning, and domestic violence. The survey involved under 5 children and men and women aged between 15 and 49 years residing in noninstitutional settings. A two-stage stratified cluster sampling design is used to draw household samples. In the first stage, clusters are selected using a probability proportional to size (PPS) sampling technique. Then, in the second stage, a fixed number of households (usually 28-30 households) are selected using a systematic sampling technique. The analysis was done on 63,266 pregnant women from individual recode (IR) file. DHS data are available in public domain and can be accessed at http://dhsprogram.com/data/available-datasets.cfm. A more detailed information about DHS data was published elsewhere [22, 23].

### 2.2. Selection of Variables and Measurement

#### 2.2.1. Outcome Variable

Outcome variable was ANC attendance. This was categorized as follows: (0) eight or more ANC attendances and (1) insufficient or inadequate ANC visits (less than eight ANC visits). For inadequate or insufficient ANC visits, the new recommended guidelines by the WHO were considered [10, 18], making less than eight visits.

#### 2.2.2. Explanatory Variables

Selection of individual and community level explanatory variables was based on a broad literature review [2, 8, 9, 14, 20, 22, 24–33] and their availability in the DHS datasets. The following variables were considered and included in the analysis.

*(1) Individual Level Explanatory Variables*. Individual level explanatory variables included women's age (15–49); women's level of education (no formal education, primary, secondary, and higher); religion (Muslim, Christian, other religion, and no religion); health insurance registration (no and yes); marital status (unmarried and married); sex of household head (male and female); parity (1-2, 3-4, and 5+); family size (<5 and 5+); currently working (no and yes); economic status (poorest, poorer, middle, richer, and richest); and media exposure (no and yes). As DHS now does not collect information on income, family wealth index was used as a proxy for economic status. It is measured mainly based on component rankings generated through principal component analysis on ownership of family assets, for example, supply of drinking water, kind of toilet facility, sort of cooking fuel, and possession of television and fridge. Based on individual rankings, households have been categorized into five classes on the wealth index: poorest, poorer, middle, richer, and richest [34, 35].

Exposure to media [newspaper, radio, or television (TV)] was assessed in terms of frequency (no exposure, less than once a week, an more than oncea week). We coded “yes” if the respondent read newspaper or listened to a radio or watched TV for at least less than once every week and “no” as otherwise.

*(2) Community Level Explanatory Variables*. Community level factors included and coded as follows: distance to health facility (big problem and not a big problem); place of residence (urban and rural); community literacy level (low, medium, and high); and community socioeconomic status (low, medium, and high). Big problem was considered if a pregnant woman reported that the distance to health centre or hospital was a big problem for her. Community socioeconomic status was computed from occupation, wealth, and education of study participants who resided in a given community. Principal component analyses were applied to calculate women who were unemployed, uneducated, and poor. A standardized rating was derived with an average rating (zero) and standard deviation [1]. The rankings were then segregated into tertile 1 (least disadvantaged), tertile 2, and tertile 3 (most disadvantaged) where the least rating (tertile 1) denoted greater socioeconomic status with the highest score (tertile 3) denoting lower socioeconomic status.

Correspondingly, for community literacy, respondents who had attended higher than secondary school were assumed to be literate while all other respondents were given a sentence to read, and they were considered literate if they could read all or part of the sentence. Therefore, high literacy included respondents who had higher than secondary education or had no school/primary/secondary education and could read a whole sentence. Medium literacy means respondents who had no school/primary/secondary education and could read part of the sentence. Low literacy means respondents who had no school/primary/secondary education and could not read at all. These were categorized into appropriate tertiles where tertile 1 (lowest score and least disadvantaged) was high community literacy, tertile 2 (medium score) was medium community literacy, and tertile 3 (highest score and most disadvantaged) was low community literacy.

### 2.3. Statistical Analyses

The analysis was conducted using Stata version-14 software (Stata Corp, College Station, Texas, USA) using the following steps. First, descriptive analysis comprising frequency distribution of respondents and noncompliance of ANC 8+ visits across explanatory variables was done. Then, Pearson's chi-square test of independence was carried out to select variables that had significant association with noncompliance of ANC 8+ visits at *p* value less than 0.5 cut point. Then, a multicollinearity test was done using variance inflation factor (VIF) for all statistically significant variables at the chi-square test, and we found no evidence of collinearity among the explanatory variables (mean VIF = 2.25, Min VIF = 1.05, Max VIF = 4.48). Lastly, four different models were constructed using a multilevel logistic regression (MLLR) method to assess whether or not the individual and community level factors had significant associations with the outcome variable (noncompliance of ANC 8+ visits). The first model was a null model, which had no explanatory variables, and it showed variance in noncompliance of ANC 8+ visits, attributed to Primary Sampling Unit (PSU). The second model called Model I incorporated only the individual level factors, and the third model (Model II) included community level factors only. The last model (Model III) comprised both the individual and community level factors.

All four MLLR models included fixed and random effects [36, 37]. The fixed effects indicated the association between the explanatory variables, and the outcome variable and the random effects signified measure of variation in the outcome variable based on PSU, which is measured by intracluster correlation (ICC) [38]. Finally, the model fitness or how the different models were fitted with the data was examined using the Akaike Information Criterion (AIC). We used “mlogit” command to run the MLLR models. Weighting was done to take into account the complex nature of DHS data while the “svyset” command was used for adjusting for disproportionate sampling and nonresponse. According to Hatt and Waters [39], pooling data can bring about broader results that are “often obscured by the noise of individual data sets.” To calculate the pooled values, further adjustment was needed to account for the variability in the number of individuals sampled in each country. This was accomplished using the weighting factor 1/(*A*∗*nc*/*nt*), where *A* is the number of countries asked a particular question, *nc* is the number of respondents for the country *c*, and *nt* is the total number of respondents over all countries asked the question.

## 3. Results

### 3.1. Pooled Prevalence of Noncompliance of ANC 8+ Visits

The results from the pooled analysis show 92.3% of pregnant women did not attend the newly WHO recommended ANC 8+ visits. As shown in Table 1, variations were observed across studied countries from the highest prevalence of noncompliance of ANC 8+ visits in Zambia (98.7%) to the lowest in Libya (73.4%) (Table 1).

### 3.2. Sociodemographic Characteristics of Respondents

Table 2 presents results on the sociodemographic and other selected characteristics of the respondents. About 9.6% of respondents were adolescents (15-19 years). More than half of the participants were living in rural areas (52.7%), were currently working (69.4%), and did not report distance to health facility as a big problem (57.5%) and exposed to media (it could be newspaper). Similarly, most of them had no registration for health insurance (98.2%) and were not exposed to media (57.0%); it could be newspaper, radio, or television for at least less than once a week. More than one-fourth (26.2%) of the participants had not attended formal education.

### 3.3. Prevalence of Noncompliance of ANC 8+ Visits across Explanatory Variables

Table 2 shows distribution of noncompliance of ANC 8+ visits across explanatory variables. There is a lower proportion of noncompliance of ANC 8+ visits among pregnant women with higher educational level (75.9%) and richest household (78.4%), compared to those with no formal education (98.1%) and those from the poorest households (99.1%), respectively. About 97.2% of pregnant women who were living in rural setting did not attend a minimum of eight ANC visits while this proportion lowered to 86.8% among pregnant women who were living in urban areas. Noncompliance of ANC 8+ visits was lower among pregnant women who were covered with health insurance (74.4%) compared to those without health insurance (92.6%) (Table 2).

### 3.4. Fixed Effects (Measure of Association)

#### 3.4.1. Individual Level Factors

The likelihood of noncompliance of ANC 8+ visits among pregnant women within the age groups of 35-39 and 40-44 years was lower (aOR =0.35, 95% CI: 0.16-0.75; aOR =0.33, 9% CI: 0.14-0.78, respectively), compared to pregnant women within the age groups of 15-19 years. Similarly, lower odds of noncompliance of ANC 8+ visits among pregnant women who were under the coverage of health insurance (aOR =0.53, 95% CI: 0.29-0.98) compared to those pregnant women without health insurance coverage. Moreover, lower odds of noncompliance of ANC 8+ visits were seen among pregnant women who were from poorer (aOR =0.36, 95% CI: 0.15-0.86), middle (aOR =0.33, 95% CI: 0.12-0.88), richer (aOR =0.26, 95% CI: 0.09-0.73), and richest (aOR =0.16, 95% CI: 0.05-0.49) households compared to pregnant women who were from poorest households. Conversely, the study shows higher odds of noncompliance of ANC 8+ visits among pregnant women with five or more ever born children (aOR =1.68, 95% CI: 1.12-2.52) compared to pregnant women with one or two ever born child/children.

#### 3.4.2. Community Level Factors

Community literacy level was found to be one significant factor. More specifically, the study shows lower odds of noncompliance of ANC 8+ visits were among pregnant women who were from medium (aOR =0.53, 95% CI; 0.31-0.89) and high (aOR =0.56, 95% CI; 0.32-0.99) community literacy level compared to pregnant women who were from low community literacy level.

### 3.5. Random Effects (Measures of Variations) Results

In Table 3, the values of AIC show that there was a substantial decrement in each of the individual alone model and community alone model over the final model, and this supports the goodness of fit of the final model developed in the analysis. And so, the complete model, which included the individual and community level factors, was selected for its importance in predicting noncompliance of ANC 8+ visits. The null model (Table 3) demonstrates that there was significant variation in the likelihood of noncompliance of ANC 8+ visits across the clusters (*σ*2 = 1.92, 1.48-2.50). The null model showed that 35% of the total variance in noncompliance of ANC 8+ visits was attributed to between-cluster variations (ICC = 0.35). The between-cluster variations decreased by 11% in Model I, from 35% in the null model to 14% in the individual level only model. From Model I, the ICC again increased by 1% in the community level factors (ICC =0.15) and again declined by 3% in the complete model (Model III, ICC = 0.12), which had both the individual and community level factors. This clarifies that the variations in the likelihood of noncompliance of ANC 8+ visits could be accredited to the variances in the clustering at the PSU (Table 3).

## 4. Discussion

The study explored the DHS datasets from countries in SSA spanning 2018 to 2020. Overall, the prevalence of noncompliance of at least eight ANC visits in the studied SSA countries was 92.3% (95% CI: 91.1%-93.3%) with the highest and lowest prevalence in Zambia (98.7%, 95% CI: 98.3%-99.1%) and Libya (73.4%, 95% CI: 70.4%-76.2%), respectively.

Several individual and community level factors are identified. More specifically, the finding from the current study shows that there were lower odds of noncompliance among older pregnant women compared to young pregnant women. Association between women's age and ANC service utilization was reported in Nepal [44], India [45], and SSA countries [46, 47]. This could be partly explained by lack of experience in pregnancy care among young pregnant women compared to older pregnant women [46].

We found that the likelihood of noncompliance of ANC 8+ visits among richest/rich pregnant women was lower compared to that among those pregnant women who were from poorest households. Economic status-related variations in ANC service uptake were also documented in Guinea [48–50]. The plausible justification for lower odds of noncompliance of recommended ANC services among pregnant women from richest/rich households could be due to their affording capacities of medical and nonmedical costs for ANC services [49]. Economic barriers might prevent utilization of ANC and other maternal services at all or reduce its qualities due to direct and indirect costs [49]. As documented in a prior study in Ethiopia, socioeconomic disparities in ANC service might be explained by the variations in women's and their husband's educational level and occupation type since it facilitates rich women to access healthcare services than the their poor counterparts [50].

We found that the likelihood of noncompliance of pregnant women with more children was higher compared to that of those pregnant women with few children. It is comparable with prior studies in Ghana and South Africa [51, 52]. Parity not only influences ANC service uptake but also eventually leads to no usage of skilled birth attendance services [53–55]. The possible justification for not using the recommended ANC services might be due to lack of time and resources caused by larger family as well as their self-confidence developed from prior pregnancy and childbirth [51, 52, 55].

We found that lower odds of noncompliance of ANC 8+ visits among pregnant women who were under health insurance coverage compared to those among pregnant women who did not get health insurance services. Comparable findings were reported in prior studies [56–58]. Health insurance is associated with utilization of ANC and health facility delivery services [56]. This is because health insurance can considerably decrease the burden of out-of-pocket (OOP) expenditure for healthcare services [59]. Reduction of payments for maternal healthcare services through health insurance improves healthcare-seeking and utilization behaviour.[57].

Furthermore, we found that there were lower odds of noncompliance of ANC 8+ visits among pregnant women who lived in communities with medium and high literacy levels. Evidence shows women living in communities with better literacy level face less challenges to access healthcare services [60]. This is usually associated with better revenue level [61]. Education is a crucial element for increasing the health and overall wellness of persons [61]. It actually aids in promoting and sustaining healthy lifestyles and positive selections, thereby enhancing human development as a whole [61]. Living in communities with better literacy level facilitates and allows access to health-related information that could empower them to exercise their choice and to be able to overcome cultural barriers of ANC service utilization [62, 63]. Education changes attitude and expectation of a women and her significant others' towards traditional gender norms and roles [63]. On the other hand, lack of education leads to poor quality interactions between a pregnant woman and service providers consequently discouraging utilization of ANC services [64].

### 4.1. Strengths and Limitations

The study used data from nationally representative samples to estimate the prevalence of noncompliance with the 2016 WHO-recommended guideline of minimum of eight ANC visits and its associated individual and community level factors using multimodel approach, which presents findings with enhanced accuracy and generalizability. Limitations of the study findings are however noted. Interpretation of the findings from this study is limited in making causal relationships between variables, as the study employed cross-sectional design in collecting data. There is also the possibility of under- or overestimation of the study parameters as data presented are self-reported which could not be independently verified. Finally, results from pooled analysis of data provide less information on the within and between country variations in factors associated with noncompliance with 8+ ANC visits.

### 4.2. Conclusion

Using DHS datasets from eight SSA countries, this study showed that the prevalence of noncompliance with the 2016 WHO recommended minimum of eight ANC visits was high. This may hinder the achievement of the Sustainable Development Goals (SDG) especially goal 3, which focuses on the wellbeing of mothers and children. Women's age, health insurance registration, economic status, and ever born children were significant individual level factors while community level literacy was an identified community level factor. To increase ANC visits, efforts should be made to encourage ANC visits among women in SSA through economic empowerment, enhancement of national literacy rate, and health insurance coverage. Furthermore, providing more attention for young pregnant women and those who with more children is important.

## Figures and Tables

**Table 1:** Studied country, year of survey, and sampled population.

Country	Survey year	Sampled population		Proportion who made less than 8 ANC visits (%), estimate (95% CI)
		Weighted Number	Weighted Percent	
Cameroon	2018/19	6,395	10.1	92.2 (91.1-93.2)
Gambia	2019/20	5,747	9.1	95.7 (95.1-96.3)
Guinea	2018	5,383	8.5	96.7 (96.1-97.3)
Libya	2019/20	4,185	6.6	73.4 (70.3-76.2)
Mali	2018	6,246	9.9	96.5 (95.8-97.1)
Nigeria	2018	21,465	33.9	79.7 (78.6-80.8)
Sierra Leone	2019	6,540	10.3	75.0 (72.9-76.9)
Zambia	2018/19	7,305	11.6	98.7 (98.2-99.0)
Total		63,266	100.00	92.3 (91.1-93.3)

**Table 2:** Sociodemographic characteristics of respondents and distribution of noncompliance of ANC 8+ visits (*N* = 63,266): evidence from eight SSA countries' DHS.

Variables	Number (weighted %)	Less than 8 ANC visit (weighted %)	*p* value
*Women's age (in years)*			<0.01
15-19	5,101 (9.55)	96.7	
20-24	13,305 (21.60)	93.0	
25-29	15,886 (26.88)	92.3	
30-34	12,584 (21.26)	91.0	
35-39	9,909 (13.50)	90.3	
40-44	4,699 (5.82)	90.8	
45-49	1,782 (1.39)	96.0	
*Women's level of education*			<0.001
No formal education	28,542 (26.16)	98.1	
Primary	13,594 (29.89)	94.5	
Secondary	18,059 (37.95)	89.1	
Higher	3,071 (5.99)	75.9	
*Economic status*			<0.001
Poorest	15,296 (20.62)	99.1	
Poorer	14,317 (22.13)	96.6	
Middle	13,303 (20.25)	94.8	
Richer	11,354 (19.89)	89.7	
Richest	8,996 (17.11)	78.4	
*Media exposure*			<0.001
No	2,581 (42.97)	97.5	
Yes	3,814 (57.03)	88.3	
*Sex of household head*			<0.001
Male	52,767 (79.59)	93.1	
Female	10,499 (20.41)	89.3	
*Parity*			<0.001
1-2	23,825 (40.30)	90.2	
3-4	18,235 (29.24)	92.4	
5+	21,206 (30.46)	95.0	
*Family size*			0.1120
<5	15,307 (21.52)	91.1	
5+	47,959 (78.48)	92.6	
*Marital status*			<0.01
Nonmarried	12,175 (38.75)	90.4	
Married	51,091 (61.25)	93.4	
*Currently working*			0.3223
No	22,522 (30.63)	92.3	
Yes	39,316 (69.37)	93.3	
*Health insurance registration*			<0.001
No	61,737 (98.23)	92.6	
Yes	1,529 (1.77)	74.4	
*Religion*			<0.001
Muslim	36,406 (27.82)	96.3	
Christian	26,072 (68.34)	90.5	
Other religion	421 (2.18)	95.4	
No religion	367 (1.67)	93.1	
*Place of residence*			<0.001
Urban	22,424 (47.29)	86.8	
Rural	40,842 (52.71)	97.2	
*Distance to health facility*			<0.001
Big problem	23,033 (42.49)	95.7	
Not a big problem	40,233 (57.51)	89.7	
*Community literacy level*			<0.001
Low	25,832 (43.24)	97.9	
Medium	20,934 (28.50)	90.8	
High	16,500 (28.26)	85.1	
*Community socioeconomic status*			<0.001
Low	38,103 (50.64)	97.3	
Medium	8,401 (19.27)	93.7	
High	16,762 (30.09)	82.9	

**Table 3:** Multilevel multivariable binary logistic regression results for noncompliance of ANC 8+ visits and its individual and community level factors: evidence from eight SSA countries' DHSs (*N* = 63,266).

Variables	Model 0	Model I	Model II	Model III
*Women's age (in years)*				
15-19		Ref		Ref
20-24		0.59 (0.30-1.16)		0.61 (0.31-1.20)
25-29		0.54 (0.27-1.05)		0.57 (0.29-1.11)
30-34		0.44 (0.21-0.93)^∗^		0.48 (0.23-1.00)
35-39		0.32 (0.15-0.68)^∗∗^		0.35 (0.16-0.75)^∗∗^
40-44		0.31 (0.13-0.73)^∗∗^		0.33 (0.14-0.78)^∗^
45-49		0.60 (0.13-2.81)		0.65 (0.14-3.01)
*Women's level of education*				
No formal education		Ref		Ref
Primary		0.77 (0.40-1.49)		0.87 (0.45-1.67)
Secondary		0.69 (0.36-1.34)		0.83 (0.43-1.58)
Higher		0.52 (0.24-1.11)		0.63 (0.30-1.33)
*Religion*				
Muslim		Ref		Ref
Christian		0.85 (0.59-1.23)		0.98 (0.66-1.44)
Other religion		0.51 (0.17-1.57)		0.60 (0.19-1.89)
No religion		0.76 (0.29-1.96)		0.84 (0.31-2.24)
*Health insurance registration*				
No		Ref		Ref
Yes		0.51 (0.28-0.95)^∗^		0.53 (0.29-0.98)^∗^
*Marital status*				
Nonmarried		Ref		Ref
Married		0.95 (0.67-1.36)		0.91 (0.64-1.29)
*Sex of household head*				
Male		Ref		Ref
Female		0.78 (0.59-1.05)		0.80 (0.60-1.07)
*Parity*				
1-2		Ref		Ref
3-4		1.33 (0.98-1.82)		1.31 (0.96-1.80)
5+		1.75 (1.17-2.63)^∗∗^		1.68 (1.12-2.52)^∗^
*Economic status*				
Poorest		Ref		Ref
Poorer		0.29 (0.12-0.68)^∗∗^		0.36 (0.15-0.86)^∗^
Middle		0.19 (0.08-0.48)^∗∗∗^		0.33 (0.12-0.88)^∗^
Richer		0.12 (0.04-0.29)^∗∗∗^		0.26 (0.09-0.73)^∗^
Richest		0.06 (0.02-0.17)^∗∗∗^		0.16 (0.05-0.49)^∗∗^
*Media exposure*				
No		Ref		Ref
Yes		0.83 (0.55-1.26)		0.94 (0.62-1.42)
*Distance to health facility*				
Big problem			Ref	Ref
Not a big problem			0.67 (0.50-0.91)^∗^	0.75 (0.55-1.02)
*Place of residence*				
Urban			Ref	Ref
Rural			1.95 (1.27-3.01)^∗∗^	1.44 (0.89-2.35)
*Community literacy level*				
Low			Ref	Ref
Medium			0.35 (0.21-0.58)^∗∗∗^	0.53 (0.31-0.89)^∗^
High			0.34 (0.20-0.58)^∗∗∗^	0.56 (0.32-0.99)^∗^
*Community socioeconomic status*				
Low			Ref	Ref
Medium			0.81 (0.48-1.34)	1.03 (0.63-1.68)
High			0.38 (0.23-0.65)^∗∗∗^	0.65 (0.37-1.13)
*Random effect*				
PSU variance (95% CI)	1.92 (1.48-2.50)	0.69 (0.47-1.01)	0.75 (0.51-1.09)	0.67 (0.45-1.01)
ICC	0.35	0.14	0.15	0.12
LR Test	218.91	40.11	43.25	30.34
Wald chi-square and p-value	Ref	*χ*^2^ = 191.24, *p* < 0.001	*χ*^2^ = 171.24, *p* < 0.001	*χ*^2^ = 258.44, *p* < 0.001
*Model fitness*				
Log-likelihood	-1608.87	-1489.67	-1518.19	-1475.21
AIC	3221.74	3027.35	3052.39	3010.43
*N*	63,266	63,266	63,266	63,266

^∗^*p* < 0.05;  ^∗∗^*p* < 0.01;  ^∗∗^*p* < 0.001; Ref: reference category; AIC: Akaike Information Criterion; PSU: primary sampling unit; *N*: total observation; LR: likelihood ratio; ICC: intraclass correlation coefficient.

## Data Availability

Data for this study were sourced from Demographic and Health Surveys (DHS) and available at https://dhsprogram.com/data/available-datasets.cfm

## Abbreviations

AIC: Akaike Information Criterion
ANC: Antenatal care
aOR: Adjusted Odds Ratio
CI: Confidence interval
DHS: Demographic Health Survey
ICC: Intraclass correlation coefficient
LMICs: Low- and middle-income countries
LR: Likelihood ratio
PSU: Primary sampling unit
SSA: Sub-Saharan Africa
WHO: World Health Organization.
